# Effects of Vegetable Proteins on Hypercholesterolemia and Gut Microbiota Modulation

**DOI:** 10.3390/nu10091249

**Published:** 2018-09-06

**Authors:** Marco Busnelli, Stefano Manzini, Cesare R. Sirtori, Giulia Chiesa, Cinzia Parolini

**Affiliations:** 1 Department of Pharmacological and Biomolecular Sciences, Università degli Studi di Milano, 20133 Milano, Italy; marco.busnelli@gmail.com (M.B.); stefano.manzini@gmail.com (S.M.); giulia.chiesa@unimi.it (G.C.); 2 Centro Dislipidemie, A.S.S.T. Grande Ospedale Metropolitano Niguarda, 220162 Milano, Italy; cesare.sirtori@icloud.com

**Keywords:** protein food group, cholesterol, microbiota, soybeans, lupins, peas, hempseed, functional food, LDL-receptor, PCSK9

## Abstract

Risk assessment tools, i.e., validated risk prediction algorithms, to estimate the patient’s 10-year risk of developing cardiovascular disease (CVD) should be used to identify high-risk people for primary prevention. Current evidence confirms that appropriate monitoring and control of risk factors either reduces the likelihood of CVD or slows down its progression. It is thus crucial that all health professionals make appropriate use of all the available intervention strategies to control risk factors: from dietary improvement and adequate physical activity to the use of functional foods, food supplements, and drugs. The gut microbiota, which encompasses 1 × 10^14^ resident microorganisms, has been recently recognized as a contributing factor in the development of human disease. This review examines the effect of both some vegetable food components belong to the “protein food group” and the underexploited protein-rich hempseed on cholesterolemia and gut microbiota composition.

## 1. Introduction

Cardiovascular disease (CVD) is the leading cause of death and disability worldwide, mostly due to ischaemic heart disease and stroke (both haemorrhagic and ischaemic). International and national policies now support targeting of interventions to reduce risk of CVD among high-risk patients. Accordingly, there is an increasing number of risk scores available to aid in the identification of individuals with a high CVD risk [1,2].

Almost these entire scores estimate personalized prognosis in terms of both absolute risk and life expectancy free of CVD. The use of these lifetime estimations has been endorsed by prevention guidelines to facilitate doctor–patient communication or cultivate patient motivation and, as a consequence, patient compliance [3,4,5].

**Figure 1:**
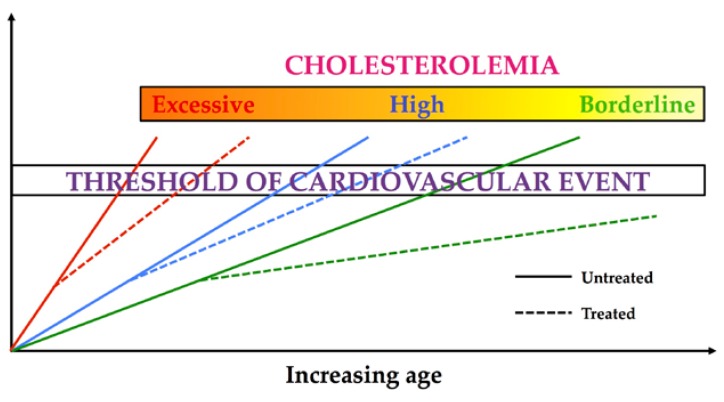
Treatment effect linked to cholesterol levels on the potential age of clinical cardiovascular event appearance. Treated means a multifactor intervention, i.e., dietary and lifestyle modifications plus the consumption of functional foods plus the use of drugs.

Consequently, appropriate monitoring and control of risk factors, carried out in a timely and continuous manner, can in fact now play an even greater role in prevention. Several randomized clinical trials and meta-analyses have shown that such management either reduces the likelihood of CVD or slows down its progression (Figure 1) [6,7,8]. Moreover, hypercholesterolemia play a key role in determining CVD and the lowering of plasma low-density lipoprotein (LDL) cholesterol (LDL-C) levels is associated with CVD risk reduction, as documented by data obtained in clinical practice [8,9,10].

It is thus mandatory that all health professionals make appropriate use of all the available intervention strategies to control risk factors: from dietary improvement and adequate physical activity (i.e., lifestyle changes) to the use of functional foods, food supplements, and drugs [8,11,12,13,14,15,16]. The Western lifestyle, including over-feeding of highly refined diets and sedentary behaviour, is associated with high prevalence of chronic conditions, such as CVD, inflammatory bowel disease (IBD) and type II diabetes, which carry a remarkable socioeconomic burden [17,18,19,20]. Proposed mechanisms range from generation of bioactive metabolites to inducing systemic low-grade inflammation.

**Figure 2:**
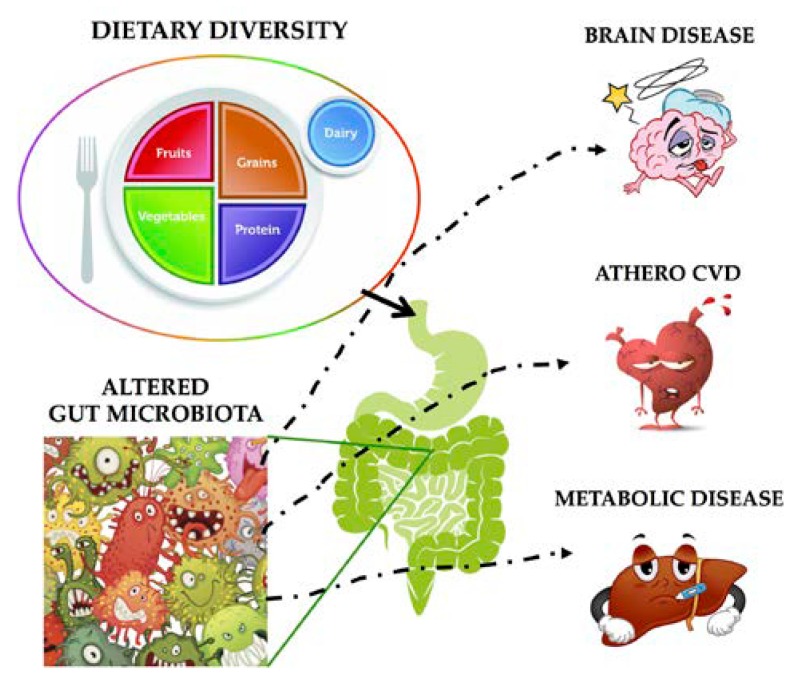
Impact of the dietary intake on gut microbiota and on human disease. Athero CVD (cardiovascular disease) means Atherosclerotic cardiovascular disease.

The gut microbiota, which includes the trillions of resident microorganisms, including bacteria, viruses, fungi, and protozoa, has been recently recognized as a contributing factor in the development of human disease [20,21]. These organisms are involved in digestion, protection against invading organisms, and regulation of metabolism and immunity. An alteration of these microbial functions has been associated with acute and chronic disease, and development of autoimmune disorders. Diet also notably has an immediate and dramatic impact on microbial structure, and may be the single most important driver of gut bacterial composition and function (Figure 2) [22,23]. The effects of high protein consumption on gut microbiota composition are not yet extensively studied, but are of increasing importance [21].

We review the effects of vegetable proteins on hypercholesterolemia and on the gut microbiota.

## 2. Plasma Cholesterol Control: From Soy to Hempseed

Recent meta-analyses have elucidated the role of diet interventions in the reduction of plasma low-density lipoprotein (LDL) cholesterol (LDL-C) levels. Many studies have found that the most commonly prescribed dietary interventions (a reduction of dietary cholesterol, and an increase in polyunsaturated fatty acids) have both a limited impact on LDL-C concentrations (about −3%) [24,25], and a low compliance to these dietary manipulations over time [8]. Moreover, the reduction of dietary saturated fats does not appear to reduce either CVD risk or all-cause mortality, even in the presence of a marked reduction of plasma LDL-C levels [26].

On the other side of the coin, other dietary protective effects that are not mediated by LDL-C variations may play a major role in CVD prevention [3]. This review will examine the effect of: (a) some old cholesterol-lowering food components belong to the “protein food group” as reported by the Dietary Guidelines for Americans 2015–2020 (U.S. Department of Health and Human Services); (b) the underexploited protein-rich hempseed [27].

### 2.1. Glycine Max

*Glycine max*, normally named as soybean, is widely cultivated for its lipid content, and indeed is the top oilseed produced worldwide [28]. In 2013, the United States accounted for about 30% of world soy production, even though historically soy consumption come from Asian countries [29]. In addition, soybean is a legume recognized as a valuable source of nutrients, i.e., they contain high-quality protein (~40%); polyunsaturated fatty acids (~18%); carbohydrates (~8%); and dietary fibres (~17%) [30].

#### 2.1.1. Experimental Evidences

Soybean protein consumption has been shown to successfully reduce cholesterolaemia in a variety of animal models [30,31,32]. In vivo and in vitro studies have attempted to establish a link between the hypocholesterolaemic effects of soybeans and the activation/depression of liver LDL receptor (LDL-R) [33,34,35]. Much of the focus on soybean has been directed toward the hypocholesterolemic properties of bioactive peptides coming from soy protein digestion, which exert their effects primarily through mechanisms involving the LDL-R, and bile acid regulation [30,36,37]. 

#### 2.1.2. Clinical Studies

Starting from the earliest studies in the Seventies, a large number of clinical studies have supported the health benefits of soybeans in humans, where elevated plasma cholesterol levels were of genetic or non-genetic origin [31,38,39]. For example, prospective observational studies in the Asian population showed that a significant reduction of total and LDL-C plasma concentrations was observed when consuming a daily amount of about 6 g of soybean protein [31]. Moreover, a meta-analysis of 38 controlled clinical trials pointed out that there is a direct correlation between soy proteins consumption (an average of 47 g/day) and the lowering of the plasma lipid levels [40].

It is important to highlight that the above mentioned cholesterol-reducing effects of soy proteins became the basis of the soy health claim relating 25 g soy protein with a reduced risk of CVD in the United States [41] and Canada [42], but not Europe (DOI: European Food Safety Authority). However, other constituents in soy have been shown to confer many health benefits, including reduction of CVD risk, and are worthy of further examination.

### 2.2. Lupinus

Lupins belong to four major species, i.e., *Lupinus albus*, *Lupinus angustifolius*, *Lupinus luteus*, and *Lupinus mutabilis*. *L. albus* and *luteus* mainly grow in the Mediterranean area, *L. angustifolius* mainly in Australia and South America, and *L. mutabilis* in the Andes. Lupin seeds are also considered very useful, from a nutritional point of view, because they contain up to 42% protein, 10% fat, 10% carbohydrates, and 30% fibre [31,43].

#### 2.2.1. Experimental Evidences

In a study performed by our group, rats were fed for 28 days Nath’s hypercholesterolaemic diets containing 20% casein or *L. angustifolius* proteins [44]. After 14 and 28 days of dietary treatment, *L. angustifolius*-fed rats markedly lowered plasma LDL-C levels compared to those measured in rats fed casein diet, (−60.1%, and −61.2%, respectively) [45]. In these animals higher hepatic mRNA levels of sterol regulatory element-binding protein-2 (SREBP-2), a major transcriptional regulator of intracellular cholesterol levels, as well as of cholesterol 7 α-hydroxylase (CYP7A1), the rate-limiting enzyme in bile acid biosynthesis, were observed, thus providing a definite mechanism underlying the plasma cholesterol concentration reduction [44,45]. In addition, in a rabbit model, lupin protein administration, compared to casein, also exerted a remarkable reduction of cholesterolemia [46,47,48]. Studies in lactating rats fed diet containing 20% of *L. angustifolius* protein markedly reduced both total cholesterol and triglyceride plasma levels [49]. A reduction of cholesterolemia and triglyceridemia were also found in apo-E deficient mice fed 10% *L. angustifolius* protein for 16 weeks [50,51,52,53].

#### 2.2.2. Clinical Studies

A randomised, double-blind, clinical study was designed with the aim at evaluating the effect of plant proteins (lupin proteins or pea proteins) and their combinations with soluble fibres (oat fibres or apple pectin) on plasma total cholesterol concentrations. Each group consumed two bars containing specific protein/fibre combinations: the reference group consumed casein cellulose. Highly significant reductions of cholesterolemia were observed in subjects receiving the bars with lupin protein + cellulose, or casein + apple pectin, or pea protein + oat fiber or apple pectin [54].

### 2.3. Pisum Sativum L.

Pea (*Pisum sativum* L.) is one of the main legumes cultivated and consumed worldwide due to its high nutritional value, low content of antinutritional substances and proven health-promoting actions [55]. Pea seeds consist of 21–22% of proteins and contain 1.5% lysine and the usefulness of pea in human nutrition is determined mainly by this high protein content and exogenous amino acids [56,57].

#### 2.3.1. Experimental Evidences

Several studies using different animal models demonstrated the impact of pea proteins on plasma lipids. In these studies, a marked hypolipidemic activity of this dietary component was observed [58,59,60,61]. A major focus of these studies was the investigation of potential mechanisms explaining the impact of pea proteins on circulating plasma total cholesterol and triglycerides. Whereas no relevant variations of SREBP-2, hydroxymethyl-glutaryl-CoA (HMG-CoA) reductase and CYP7A1 were observed, the LDL-R expression was significantly elevated in pea protein-fed animals compared with controls [59,61,62,63]. 

#### 2.3.2. Clinical Studies

Peas, as well as dietary non-oil-seed pulses have received particular attention for their ability to reduce the risk of cardiovascular disease. Their consumption was associated with a reduction in cardiovascular disease [64] and with improvements in LDL-C levels in observational trials [65,66,67]. 

### 2.4. Cannabis sativa L.

The great interest for hempseed (*Cannabis sativa* L.) depends on its nutritional content (whole seed): 35.5% oil, 24.8% protein, 20–30% carbohydrates, 27.6% total fiber (5.4% digestible and 22.2% non-digestible fiber), and 5.6% ash. Moreover, the concentration of the main anti-nutritional factors, such as phytic acid, condensed tannins, and trypsin inhibitors, is low [68]. The seed of the non-drug cultivar of industrial hemp is an underexplored protein source. The use of hempseed, as human food, dates back probably to pre-history, also with fiber utilization as textile. Cultivation of this plant has been banned for some decades in many developed countries because of the morphological similarity with marijuana. Cultivation of non-drug cultivars of industrial hempseed has become legal again in recent years because of the prevalence of low Δ9-tetrahydrocannabinoil (THC) cultivars (THC content < 200 mg/kg) [68].

#### 2.4.1. Experimental Evidences

Hempseed proteins mainly consist of a storage protein, edestin, accounting for 60–80% of total protein content, with albumin accounting for the rest. Hydrolysis of hempseed proteins allowed the identification of a number of peptides mainly belonging to edestin 1, 2, and 3, and also to other protein families [69]. Zanoni et al. by HPLC ESI-MS/MS analyses identified 90 peptides from 33 proteins. These hydrolysates showed that the highest number of active peptides was from 6 isoforms of edestin 1, other peptides belonged to well-known proteins characteristic of plants. Hempseed peptides did not impair HepG2 cell viability and, at concentrations between 0.1 and 1 mg/mL, showed a powerful activity on HMG-CoA reductase [69]. Moreover, exposure to hempseed peptides raised LDL-R activity and LDL uptake at concentrations above 0.5 mg/mL. Uptake was raised by 200% by hempseed peptides at concentrations of 0.5 mg/mL. Interestingly, hempseed peptides at concentrations of 0.5 mg/mL or higher also markedly raised proprotein convertase subtilisin/kexin type 9 (PCSK9) protein levels, thus resembling the activity of statins, i.e., reducing cholesterol synthesis and raising LDL-R and PCSK9 protein levels [70,71]. A more recently study showed the production of a high number of peptides, in the weight range of 1500–2100 Da [72]. A number of peptides showed additional bioactivities, particularly edestin 2 also added a significant antagonism to the angiotensin converting enzyme (ACE) as well as a glucose uptake stimulating activity [72].

#### 2.4.2. Clinical Studies

No clinical data on lipid changes are available up to now.

## 3. Gut Microbioma Modulation: From Soy to Hempseed

### 3.1. Glycine Max

Several recent studies have reported that the consumption of soybean or soy foods may alter the composition and population of the gut microbiota [73,74]. Efforts have also been made to elucidate the precise components in soybean that may contribute to modulation of the gut microbiota. It has been hypothesized that soybean protein can serve as nitrogen and energy sources for bacteria, which support their growth and maintenance in the gut [75,76].

An and colleagues observed a shift of bacteria composition in the Firmicutes phylum, specifically an increase in the abundance level of Enterococcus and decreased levels of Ruminococcus and Lactobacilli, after 16 days of supplementation of 20% soybean protein in a Wistar rat model [77]. Butteiger and colleagues supplemented soy protein concentrates in a Western style diet for 3 weeks and observed significant increases in Bifidobacteriaceae, Clostridiales, and Deferribacteraceae and decreases in Bacteroidetes in a Golden Syrian hamster model [74].

The major bacteria that can metabolize protein in the gut are Clostridium and Bacteroides [78]. Differential changes of bacteria in Clostridium and Bacteroides genera upon soy protein consumption may indicate a complex interaction between soy protein and gut bacteria, as well as among the bacteria in the gut. Significantly higher levels of Bacteroides and Prevotella were observed in individuals consuming soy milk containing 49.5% β-conglycinin and 6% glycinin compared to those consuming soy milk with 26.5% β-conglycinin and 38.7% glycinin [73], indicating that a higher β-conglycinin to glycinin ratio in protein content may preferentially promote the growth of bacteria in the Bacteroidetes phylum [28].

### 3.2. Pisum Sativum L.

As nutritional substrates, proteins are prone to spontaneously undergo non-enzymatic glycosylation (glycation), which can alter their molecular structure, making them highly bioactive. Glycated food proteins are able to modify the bacterial intestinal ecosystem, which is of great importance for the optimal usage of nutrients and maintenance of both intestinal homeostasis and balanced health status of the consumer. Due to their high lysine content, the pea proteins are susceptible to spontaneous glycation during storage and cooking [79].

The impact of glycated pea proteins on the intestinal bacteria from a healthy human was investigated using a protocol aimed to better understand the impact of glycated food products on homeostasis of the gut ecosystem of healthy persons. The glycated pea proteins affected the growth of gut commensal bacteria, particularly Lactobacilli and Bifidobacteria, whose levels increased significantly. There was a corresponding shift in the bacterial metabolites with increased levels of the short chain fatty acids, i.e., acetate, propionate lactate, and butyrate. Moreover, intestinal bacteria were able to utilize these pea proteins, indicating that the energy encrypted in these glycated proteins, partially inaccessible for gastric enzymes, may be recovered by gut microbiota [79]. The results obtained in this study expand our current knowledge of the interactions between glycated food proteins and gut microbiota.

In addition, glycated pea proteins were shown to beneficially modulate bacterial adhesion to enterocytes as well as its profile [80]. Such changes in microbial composition may beneficially impact the intestinal environment and exert a health-promoting effect in humans. However, further research on the interaction between glycated proteins and the human digestive system is required to determine their beneficial nutritional effect.

Unfortunately, no data are yet available showing a direct activity of lupin and hempseed proteins on gut microbiota.

## 4. Conclusions

In summary, we attempt to summarize the current knowledge of the effects of vegetable bioactive components on hypercholesterolemia and gut microbiota. On the basis of the existing literature, it is manifest that vegetable proteins, beyond the cholesterol-lowering effects, can potentially modulate gut microbiota. In most cases, the shift in gut microbiota composition, such as increases in probiotics (Lactobacilli and Bifidobacteria) and the Firmicutes to Bacteroidetes ratio, favours a health-promoting role of soybean and glycated pea proteins. However, several critical issues need to be addressed for potential future directions.
